# Coexistent Pseudogout and* Mycobacterium avium-intracellulare* Septic Arthritis in a Patient with HIV and ESRD

**DOI:** 10.1155/2016/5495928

**Published:** 2016-10-10

**Authors:** Wais Afzal, Omer M. Wali, Kelly L. Cervellione, Bhupinder B. Singh, Farshad Bagheri

**Affiliations:** Departments of Internal Medicine and Clinical Research, Jamaica Hospital Medical Center, 8900 Van Wyck Expressway, Jamaica, NY 11418, USA

## Abstract

Pseudogout is a crystal-induced arthropathy characterized by the deposition of calcium pyrophosphate dihydrate (CPPD) crystals in synovial fluid, menisci, or articular cartilage. Although not very common, this entity can be seen in patients with chronic kidney disease (CKD). Septic arthritis due to* Mycobacterium avium-intracellulare* (MAI) is a rare entity that can affect immunocompromised patients such as those with acquired immunodeficiency syndrome (AIDS) or those who are on immunosuppressive drugs. Here, we describe a 51-year-old female who presented with fever, right knee pain, swelling, warmth, and decreased range of motion for several days. The initial assessment was consistent with pseudogout, with negative bacterial and fungal cultures. However, due to high white blood cell (WBC) count in the synovial fluid analysis, she was empirically started on intravenous (IV) vancomycin and piperacillin-tazobactam and discharged on IV vancomycin and cefepime, while acid-fast bacilli (AFB) culture was still in process. Seventeen days later, AFB culture grew* Mycobacterium avium-intracellulare* (MAI), and she was readmitted for relevant management. This case illustrates that septic arthritis due to MAI should be considered in the differential diagnosis of septic arthritis in immunocompromised patients.

## 1. Introduction

Patients with CKD are prone to develop various articular pathologies including osteodystrophy, osteonecrosis, dialysis-related amyloidosis, septic arthritis, malignancy, and crystal-induced arthropathies [1]. Pseudogout is a crystal-induced arthropathy characterized by the deposition of CPPD crystals in synovial fluid, menisci, or articular cartilage. The likely mechanism behind pseudogout in CKD is secondary hyperparathyroidism, which is due to increased resistance to the parathyroid hormone (PTH) [2]. Joint aspiration and observation of positively birefringent rhomboid-shaped crystals under polarized light microscopy will establish the diagnosis.

Septic arthritis due to MAI is a rare entity that most commonly occurs in immunocompromised patients such as advanced human immunodeficiency virus (HIV) patients or those who are on immunosuppressive drugs [3–5]. In advanced HIV patients, MAI infection typically develops in generalized fashion; however, rarely isolated bone and joint infection can occur [3]. MAI-related septic arthritis results from percutaneous inoculation or hematogenous seeding [4]. Clinical history and physical examination, as well as basic lab work, raise suspicion; nevertheless, definitive diagnosis is established by the joint aspiration, biopsy, and microbiologic cultures. Polymerase chain reaction (PCR) is also considered as an adjuvant diagnostic modality [6]. Treatment in the majority of cases consists of surgical debridement and antimycobacterial drugs [6].

According to our knowledge, this is the first case illustrating coexistent pseudogout and MAI arthritis to be reported in the literature. Here, we present a case of a patient with advanced HIV infection on highly active antiretroviral therapy (HAART) with end-stage renal disease (ESRD) on hemodialysis who presented with fever, right knee joint pain, swelling, warmth, and stiffness. She was ultimately found to have culture-proven MAI arthritis.

## 2. Case Report

A 51-year-old African American female presented to the emergency department of our hospital with fever of 101°F (38.3°C), right knee pain, swelling, warmth, and decreased range of motion for several days which began after undergoing left heart catheterization one week priorly in which the right femoral artery and right femoral vein were accessed. A diagnosis of Takotsubo cardiomyopathy was established at that time. She did not report any other history of trauma. She reported essential hypertension, diabetic mellitus type 1, AIDS diagnosed in 2010 on HAART, ESRD on hemodialysis, and recent Clostridium difficile infection. There was no personal or family history of rheumatologic diseases. A review of medications indicated that she was on low dose aspirin, amlodipine, metoprolol, Insulin Glargine (Lantus®) with regular insulin sliding scale, cinacalcet, renal vitamin (Nephrocaps®), erythropoietin during hemodialysis sessions, folic acid, pantoprazole, atovaquone, darunavir, raltegravir, ritonavir, and oral vancomycin. Physical examination revealed edema, warmth, tenderness to palpation, and decreased passive range of motion of the right knee. All other joints including right ankle and right hip were normal.

Laboratory findings indicated WBC of 7600/*μ*L (reference range of 4000–10,000/*μ*L) of which 66.5% were neutrophils and 23.2% were lymphocytes. Chemistry showed random blood glucose of 335 mg/dL (reference range of <140 mg/dL), blood urea nitrogen (BUN) of 51 mg/dL (reference range of 8–20 mg/dL), creatinine (Cr) of 9.2 mg/dL (reference range of 0.7–1.3 mg/dL), potassium (K^+^) of 5.7 mEq/L (reference range of 3.5–5 mEq/L), aspartate aminotransferase (AST) of 49 units/L (reference range of <35 units/L), alanine aminotransferase (ALT) of 99 units/L (reference range <35 units/L), and alkaline phosphatase (ALP) of 173 units/L (reference range of 36–92 units/L).

X-rays of the right knee did not reveal any fractures, bony malalignment, joint space narrowing, or effusion; however, patchy vascular calcifications of the tibial artery and pretibial space were seen. She was admitted and she underwent arthrocentesis of the right knee from a medial approach. A 44 mL sample of straw-colored, turbid fluid was aspirated and sent to the lab for analysis and cultures. WBC was 250,000/*μ*L (95% neutrophils), and red blood cell (RBC) count was 50,000/*μ*L. Positively birefringent rhomboid-shaped crystals were also reported. Blood cultures were sent. Uric acid, erythrocyte sedimentation rate (ESR), C-reactive protein (CRP), anti-nuclear antibody (ANA), and Lyme antibody, as well as gonococcal and chlamydial urine DNA probes, were ordered. Meanwhile, she was started on renally adjusted dose of colchicine for pseudogout. Results returned as uric acid 7.5 mg/dL, ESR 133 mm/hr, CRP 29.6 mg/dL, negative ANA, negative Lyme antibody, and negative DNA probes for gonorrhea and chlamydia. Blood cultures did not grow any microorganisms.

A CT without contrast of the right knee was performed which revealed no fractures or evidence of necrotizing fasciitis. However, soft tissue edema of the right knee joint was observed. Bedsides, the right lower extremity Doppler was done and was inconsistent with deep vein thrombosis. At this time, operative debridement was recommended, as well as IV vancomycin and piperacillin-tazobactam; the latter was later on switched to IV cefepime.

HAART which was held on admission was resumed. CD4 helper T cell counts were reported as 146 cells/*μ*L (reference range of 490–1700 cells/*μ*L), and CD4/CD8 ratio was of 0.08 (reference range of 0.86–5). Her most recent HIV viral load performed four years earlier was 537882 copies/mL.

She underwent right knee arthroscopy under spinal anesthesia. Irrigation and debridement and partial synovectomy were performed. Samples were sent for analysis and cultures. Orthopedic diagnosis based on direct visualization of knee joint through arthroscopy was proposed as possible septic arthritis, lateral meniscal fraying, and grade II-III chondromalacia of distal patella.

Initial bacterial culture results were reported as negative. No acid-fast bacilli were seen. However, AFB culture was still in process. Final bacterial and fungal cultures were negative. Due to high suspicion of septic arthritis, she was discharged with a four-week course of IV vancomycin and cefepime to subacute rehabilitation.

Seventeen days after arthroscopy, microbiology lab reported that the synovial fluid culture using the MGIT 960 Liquid Culture method and Epicenter® program grew* Mycobacterium avium-intracellulare*. In order to decrease the chances of contamination, signal-positive tubes were subcultured on tryptase soy agar plates with 5% sheep blood added and the growth of MAI was confirmed. The acid-fast stain was performed on smears obtained from culture and was positive. We did not utilize PCR for confirmation.

She was readmitted, IV vancomycin and cefepime were discontinued, and she was started on a combination of azithromycin, ethambutol, and ciprofloxacin. MRI of right lower extremity without contrast was performed, and the report was consistent with subcutaneous and intramuscular edema surrounding the right knee. However, no bony involvement was seen. Three days later, she was discharged back to subacute rehabilitation with four weeks of treatment and was told to continue HAART and atovaquone instead of trimethoprim-sulfamethoxazole (she had sulfa allergy) for* Pneumocystis jiroveci* prophylaxis. Given her very low CD4 count and no up-to-date HIV viral load, she was advised to follow-up with infectious disease clinic upon discharge from subacute rehabilitation. Unfortunately, she did not follow-up. Multiple attempts by social worker were made to locate her and encourage her to follow-up; however, neither could she be reached by telephone nor located in previous address present in the chart.

## 3. Discussion

Articular involvement such as osteodystrophy, osteonecrosis, dialysis-related amyloidosis, septic arthritis, malignancy, and various crystal-induced arthropathies is common in patients with CKD [1]. Although not very common, pseudogout, which is a crystal-induced arthropathy, can be seen in patients with CKD as well [7]. One study in the United Kingdom revealed a close relationship between CKD and pseudogout (OR 2.29, 95% CI 1.31, 4.01) [8]. It has been suggested that parathyroid hormone-induced calcium and pyrophosphate immobilization and concentrations may lead to CPPD deposition and pseudogout [8]. Pseudogout usually affects one or multiple large joints and manifests as severe arthralgia, warmth, erythema, and edema due to effusion. Diagnosis is established by the joint aspiration and detection of positively birefringent rhomboid-shaped crystals under polarized light microscopy. These findings were very similar and consistent in our patient as well. However, her symptoms did not abate with colchicine. Broad spectrum antibiotics for possible infectious etiology also did not provide sufficient relief. Partial pain and edema improvement could be attributed to incision and drainage. A satisfactory answer was achieved upon the growth of MAI in the culture 17 days later.

MAI typically causes disseminated disease with overt systemic symptoms in immunocompromised patients, for instance, advanced HIV patients or those on immunosuppressive drugs. The first case of MAI septic arthritis was reported in 1976 in a young patient receiving chemotherapy [9]. Septic arthritis due to MAI is considered a rare entity, but it can occur in immunocompromised patients [3–5]. Our patient was also severely immunocompromised as indicated by her low CD4 counts and CD4/CD8 ratio. There are case reports indicating MAI as the cause of septic arthritis in immunocompetent hosts as well [10–12]. However, the number of MAI-induced septic arthritis in immunocompetent patients is by far less common compared to immunocompromised patients.

The source of MAI is considered to be soil and dust. This bacterium is introduced by percutaneous route as in trauma or microtrauma, inhalation, or ingestion which later on may spread via lymphatic or hematogenous channels. Lymphatic channels are the source of MAI reservoir in HIV patients and often presents with mesenteric lymphadenopathy. In a retrospective study of 24 HIV patients with MAC, 10 patients (42%) were found to have intra-abdominal mesenteric and retroperitoneal lymphadenopathy, and, in 5 patients (25%), it was present on CT abdomen [13]. All of those 24 patients had positive blood cultures for MAI. In our patient, the ultimate mycobacterial blood culture results reported as negative; nevertheless, in light of her very low CD4 count, there is a very high possibility that the infection would have spread hematogenously, and intra-abdominal lymph nodes would have been certainly involved. However, we did not perform a CT scan of the abdomen in our patient.

MAI, just like any other mycobacteria, has a very slow growth process and an average of a 6-month lag between trauma and clinical presentation would be expected [14]. Thus, we can confidently say that the cardiac catheterization one week before her presentation is not the cause. The pathologic hallmark of MAI infection is the formation of granuloma. Bone and joint infections due to MAI usually have an indolent course. MAI-related joint and bone infection should raise suspicion mainly when routine cultures fail to delineate an organism but histology reveals the presence of granuloma [3]. In MAI-related septic arthritis, inflammatory markers may be elevated, but they are not very specific. Synovial fluid findings are very similar to* Mycobacterium tuberculosis* arthritis. PCR is also considered to be of great reliability in diagnosing MAI [6]. Nevertheless, definitive diagnosis is established by histology and culture [3]. The management of MAI septic arthritis is comprised of surgical debridement and antimycobacterial drugs [6, 14]. Most regimens consist of macrolide and ethambutol, combined with fluoroquinolone or rifamycin. Amikacin may be considered as an add-on agent. Rifamycins are good drugs with a relatively safer profile; however, their interaction with ritonavir-booster HIV regimen remains a challenge [6]. We started our patient on azithromycin, ethambutol, and ciprofloxacin. Unfortunately, we could not track her to assess the efficacy of this regimen.

In 1997, a case of coexistent gout and MAI in a renal transplant patient was reported [15]. According to our knowledge, ours is the first case revealing coexistent pseudogout and MAI arthritis in the same patient to be reported in the literature.

There is no causal association between pseudogout and MAI arthritis. In general, joints affected by crystal-induced arthropathies have a higher predilection to become infected in comparison to normal joints [16]. This is especially true if the host is immunocompromised like our patient.
